# Combined bulk RNA-seq and single-cell RNA-seq identifies a necroptosis-related prognostic signature associated with inhibitory immune microenvironment in glioma

**DOI:** 10.3389/fimmu.2022.1013094

**Published:** 2022-11-17

**Authors:** Sicheng Wan, Ulrich Aymard Ekomi Moure, Ruochen Liu, Chaolong Liu, Kun Wang, Longfei Deng, Ping Liang, Hongjuan Cui

**Affiliations:** ^1^ The State Key Laboratory of Silkworm Genome Biology, College of Biotechnology, Southwest University, Chongqing, China; ^2^ Cancer Center, Medical Research Institute, Southwest University, Chongqing, China; ^3^ The Ninth People’s Hospital of Chongqing, Affiliated Hospital of Southwest University, Chongqing, China; ^4^ Department of Neurosurgery, Chongqing Children’s Hospital, Chongqing, China

**Keywords:** necroptosis-related gene, necroptosis-related prognostic signature, glioma, tumor immune microenvironment, single cell RNA seq

## Abstract

Necroptosis is a programmed cell death playing a significant role in cancer. Although necroptosis has been related to tumor immune environment (TIME) remodeling and cancer prognosis, however, the role of necroptosis-related genes (NRGs) in glioma is still elusive. In this study, a total of 159 NRGs were obtained, and parameters such as mutation rate, copy number variation (CNV), and relative expression level were assessed. Then, we constructed an 18-NRGs-based necroptosis-related signature (NRS) in the TCGA dataset, which could predict the patient’s prognosis and was validated in two external CGGA datasets. We also explored the correlation between NRS and glioma TIME, chemotherapy sensitivity, and certain immunotherapy-related factors. The two necroptosis-related subtypes were discovered and could also distinguish the patients' prognosis. Through the glioblastoma (GBM) scRNA-seq data analysis, NRGs’ expression levels in different GBM patient tissue cell subsets were investigated and the relative necroptosis status of different cell subsets was assessed, with the microglia score culminating among all. Moreover, we found a high infiltration level of immunosuppressive cells in glioma TIME, which was associated with poor prognosis in the high-NRS glioma patient group. Finally, the necroptosis suppressor CASP8 exhibited a high expression in glioma and was associated with poor prognosis. Subsequent experiments were performed in human glioma cell lines and patients' tissue specimens to verify the bioinformatic analytic findings about CASP8. Altogether, this study provides comprehensive evidence revealing a prognostic value of NRGs in glioma, which is associated with TIME regulation.

## Introduction

Glioma, a malignant central nervous system (CNS) tumor originating from the glial, is featured with high recurrence and poor prognosis. According to the World Health Organization (WHO), glioma classification relies on different histopathological subtypes and is classified into four grades (I-IV), with grades I-III being the low-grade glioma (LGG) and grade IV representing the aggressive form, GBM (1, 2). Although conventional surgical resection, chemoradiotherapy combined with immunotherapy and electric field therapy improve the prognosis of glioma patients to an extent, the overall prognosis of glioma patients remains poor due to the glioma heterogeneity and epigenetic mutations of intratumoral molecules (including isocitric dehydrogenase (IDH) and epidermal growth factor receptor mutations, 1p19q co-deletion, and MGMT promoter methylation). Accordingly, it is urgent to comprehensively understand the molecular mechanisms underlying glioma recurrence and progression, and discover new biomarkers for a better diagnosis and treatment of this disease (3).

Necroptosis is a novel form of regulated necrosis. It is a cell death pattern originally programmed to protect the host against microbial agents when the caspase-dependent apoptosis pathway is blocked by pathogens. Generally, the classical necroptotic pathway is triggered by extracellular stimuli, such as tumor necrosis factor (TNF), which activates the downstream receptor-interacting serine/threonine kinases 1 and 2 (RIPK1/2), leading to the phosphorylation of the mixed lineage kinase domain-like pseudokinase (MLKL). The latter then translocates to the cytoplasmic membrane to generate the pore complex, resulting in the release of damage-associated molecular patterns (DAMPs) and cellular contents, and membrane rupture (4, 5). Caspase-8 (CASP8) is a necroptosis suppressor that inhibits the necroptotic pathway by cleaving RIPK1 and RIPK3 (6). The two latter molecules show low expression level in multiple cancer types and the conventional perspective reckon that the related activated necroptosis promotes cell necrosis and leads to tumor inhibition. However, recent studies have suggested that necroptosis might play a dual role in tumors. For instance, necroptosis-mediated dying tumor cells induce the C-X-C motif chemokine ligand 1 (CXCL1) and sin3A-associated protein 130 (SAP130) release to aggravate inhibitory TIME (7, 8). Besides, DAMPs released from damaged or dying cells can promote immunosuppressive cell accumulation in the TIME (9).

In this study, we aimed to comprehensively analyze the expression patterns of NRGs in glioma, construct an NRS to predict the prognosis of glioma patients, and explore the relationship between necroptosis and glioma immune microenvironment at a single-cell level. We found the dysregulation of the necroptosis pathway in glioma and certain NRGs displayed abnormal expression, multitype mutations, and CNVs, and certain of the NRGs were associated with TIME regulation. Next, we constructed and validated an NRS that could effectively predict the prognosis and chemosensitivity of patients with glioma. And then, based on the NRS, we explored the immunotherapy difference between the two NRS groups and further discovered two necroptosis-related subtypes, which can also distinguish the patients’ prognosis. Later, we also assessed the expression of NRGs in different cell subsets at a single-cell level. Finally, we analyzed the core intersection gene *CASP8* from the perspectives of immune checkpoints, immune cell infiltration, prognosis, and protein expression, which unveiled that CASP8 can be used as a novel potential glioma prognosis biomarker.

## Materials and methods

### Datasets acquisition and processing

The expression profile with the Toil RNA-seq recompute and related clinical sample information LGG, GBM, and normal brain tissue samples) were downloaded from The Cancer Genome Altas (TCGA) [TCGA-LGG, TCGA-GBM and the Genotype-Tissue Expression (GTEx) database (UCSC Xena repository, an online cancer database designed by the University of California–Santa Cruz, http://xena.ucsc.edu/)] (10). Thereafter, LGG and GBM RNA-seq data were merged, and totals of 642 glioma samples (LGG, n=499 and GBM, n=143) and 1259 normal brain tissue samples were included in the present study.

For dataset validation, gene expression profile data (mRNAseq-693 and mRNAseq-325) and corresponding clinicopathological information for glioma patients were retrieved from the Chinese Glioma Genome Atlas (CGGA, http://www.cgga.org.cn/) (11).

Corresponding GBM scRNA-seq data from a previous scRNA-seq research (single-cell transcriptome profiles in 10 primary IDH^wt^ type GBM patients, GSE173278) were downloaded from the Gene Expression Omnibus (GEO, http://www.ncbi.nlm.nih.gov/geo/). The R-package (Seurat 4.1.0) was used to analyze scRNA-seq data (12–15). The top 20 principal components were used to construct the SNN graph and UAMP embedding. The R-package (harmony 0.1.0) was used for batch correction and cell annotation was performed based on singleR and manual cell type annotation.

### Differentially expressed NRGs identification

NRGs differential expression analysis was performed with FPKM and Wilcoxon rank sum test by using R-package (Limma) (16). Gene expression was considered significant when meeting the following criteria: adjusted *p-*value<0.05 (BH method) and |log_2_(Fold Change)| >1.0.

### Analysis of NRGs mutation

NRGs mutation frequency and oncoplot waterfall plot were generated by R-package (maftools) (17). For the gene CNVs, the value >0.2 was defined as “gain” and the value< -0.2 was defined as “loss”.

### Identification and validation of the NRS

A total of 159 NRGs were obtained from the KEGG necroptosis pathway [https://www.kegg.jp/entry/map04217) (18). By using the R-package (survival (3.2-13)], we performed the univariate Cox regression analysis to identify the NRGs related to glioma patients’ overall survival (OS) in the TCGA training set (p<0.05). A total of 126 genes were screened as potential risk factors related to the OS. Then, the LASSO regression algorithm was performed by using the R-package (glmnet) to calculate regression coefficients to further refine the gene set (19, 20). Finally, 15 NRGs were identified as the most valuable OS genes and based on normalized gene expression values and coefficients, each sample risk score was calculated using the following formula:

$\begin{array}{l} Riskscore=\sum_{i=1}^{n}(\text{exprgenei x coefficient genei}) \end{array}$

Based on the median value of all patients’ risk scores in the TCGA training set, samples were classified into high- and low-risk cohorts. For the signature validation, the same calculation as described above was employed in two CGGA (mRNAseq-693, mRNAseq-325) validation sets, respectively.

Then, we used R-packages [survival (3.2-13)” and “survminer (0.4.9)] to analyze the survival of two risk groups through the Kaplan-Meier (K-M) curve. The Log-Rank test was conducted to assess survival differences between the two groups. The time-dependent receiver operating characteristic (ROC) curve was plotted by using R-packages [timeROC (0.4)” and “survival (3.2-13)] to evaluate the predictive ability of the NRS for 6 months, 1-, 2-, and 3-year glioma patient survival rates.

### Functional enrichment analysis

To clarify the risk score-related to biological functions and pathways, the differentially expressed genes (DEGs) between high and low-risk groups in the training set were identified as described above. DEGs’ biological functions and pathways were explored through the gene ontology (GO) and Kyoto Genome Encyclopedia (KEGG) pathway enrichment analysis by using R-package (ClusterProfiler), the FDR *p*-value<0.05 was used as the cut-off criterion.

The gene set enrichment analysis (GSEA) was used to identify and compare the different cancer hallmarks between high and low-risk groups in the TCGA cohorts (https://www.gsea-msigdb.org/gsea/index.jsp).

### Immune cell infiltration analysis

To explore the relationship between the immune cell infiltration and calculated risk score, the correlation between NRS groups and different immune cell infiltration was analyzed by using the CIBERSORT, GSVA, and XCELL methods (21–23). The gene set of immune cell types was obtained from a previous research (24).

### Chemotherapeutic drug response analysis

The R-package (oncoPredict) was used to assess the drug response differences between risk score and corresponding drugs derived from the Genomics of Drugs Sensitivity in Cancer (GDSC), Cancer Therapeutics Response Portal (CTRP), and Cancer Cell Line of Encyclopedia (CCLE). Pearson coefficient was used to calculate the correlation between signature score and area under the dose-response curve (AUCs) values.

### Construction of an NRS-based nomogram

The R-package (rms) was used to build the NRS-based nomogram to predict glioma patients’ 6-months, 1- and 2-year survival probability. To validate the nomogram, the calibration was plotted, which can assess the nomogram prognostic accuracy; the 45° line represents the best prediction. The decision curve analysis (DCA) curve, drawn by R-package (rmad), was used to test the nomogram value for clinical application.

### The exploration of new necroptosis subtypes

Based on the 18 NRGs, the consensus non-negative matrix factorization (CNMF) algorithm was performed to identify new necroptosis subtypes in the TCGA glioma cohort by using the R-package (CancerSubtype). We used the silhouette coefficient to evaluate the most optimal cluster number.

### Cell culture

Human glioma (LN-229, U87-MG, U118-MG, U251-MG, and A172) and human astrocyte NHA cell lines were obtained from American Type Culture Collection (ATCC, Beijing, China). All cell lines were regularly tested for mycoplasma contamination, and cultured in Dulbecco’s modified Eagle’s medium (DMEM) (Gibco, New York, NY, USA), supplemented with 10% fetal bovine serum (Gibco, New York, NY, USA).

### Antibodies

The CASP8 (13423-1-AP) and CD11B/ITGAM (#66519-1-Ig) antibodies were purchased from Proteintech Group (Wuhan, China), and α-Tubulin (ab7291) antibody was purchased from Abcam (Cambridge, UK).

### Western blot (WB) assay

Cells were lysed with the cell lysis buffer (Beyotime). WB assay was performed as previously described (25).

### Patient specimen immunohistochemistry (IHC) assay

15 pairs of glioma patients’ tissue samples were obtained from the Affiliated Hospital of Southwest University (The Ninth People’s Hospital of Chongqing), and patients agreed and signed consent. As previously described, paraffin sections were dewaxed, hydrated, and repaired with corresponding antigens (Sangon, Shanghai China), followed by overnight incubation with CASP8 antibody (26). Visualization was made by using a horseradish peroxidase detection system.

### Immunofluorescence histochemistry (IFH) assay

After dewaxing, samples were incubated with 10 mM citric acid (pH6.0) for antigen repair and blocked with goat serum for 2 hours. Following primary antibody staining, PBS was washed and incubated with fluorescent secondary antibody for 1 hour, then washed with phosphate-buffered saline (PBS). Then, we repeated the above procedure for the second primary antibody staining. Finally, the tissue slices were mounted with medium containing 4’,6-diamidino-2-phenylindole (DAPI). Stained slides were observed by using confocal fluorescence microscopy (Leica, Germany).

### Data statistics and analysis

The bioinformatics statistics analysis was accomplished by R (version 4.1.2, Institute for Statistics and Mathematics, Vienna, Austria; https://www.r-project.org). The correlation analysis was conducted by Spearman correlation analysis. The Chi-square test was used to compare the different clinical indicators. Survival status was evaluated by Cox regression analysis and the OS, DSS, and PFS were generated by the Kaplan–Meier method and evaluated by the log-rank test, respectively. The log-rank test was used to assess the difference in immune infiltration and drug response among different NRS groups.

As for the part of experiment validation, all observations were confirmed by at least three independent biological replicates. The results in this study were presented as the means ± standard deviation (SD). Two-tailed Student’s t test was performed for paired samples. P<0.05 was considered statistically significant.

## Results

### The necroptosis pathway is dysregulated in glioma

**Figure 1:**
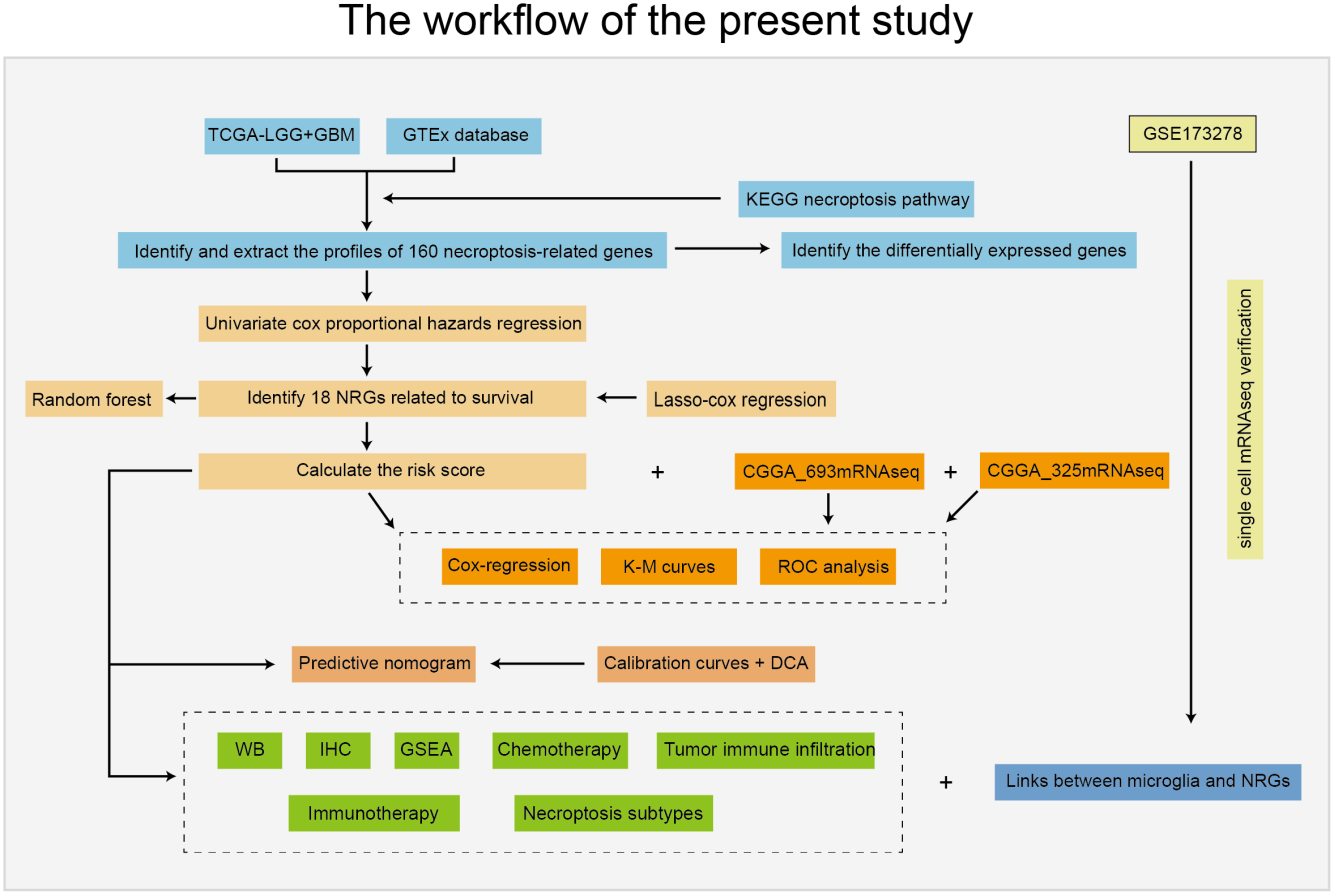
Workflow used in this study.

The workflow of this study is depicted in **Figure 1**. Patients with incomplete clinical information were excluded from the bulk RNA-seq data, and the clinical features of all included patients are summarized in **Table 1**.

**Table 1:** Patients’ clinical features from the bulk RNA-seq.

	TCGA (N = 642)	CGGA_693 (N = 692)	CGGA_325 (N = 321)	Overall (N = 1655)
**Cancer type**				
GBM	143 (22.3%)	249 (36.0%)	139 (43.3%)	531 (32.1%)
LGG	499 (77.7%)	443 (64.0%)	182 (56.7%)	1124 (67.9%)
**Age**				
Mean	46.7 (15.1)	43.3 (12.4)	43.0 (12.0)	44.5 (13.5)
Median	45.5 [14.0,89.0]	43.0 [11.0,76.0]	42.0 [8.0,79.0]	43.0 [8.0,89.0]
Missing	0 (0%)	1 (0.1%)	0 (0%)	1 (0.1%)
**Gender**				
Female	271 (42.2%)	294 (42.5%)	122 (38.0%)	687 (41.5%)
Male	371 (57.8%)	398 (57.5%)	199 (62.0%)	968 (58.5%)
**Grade**				
II	241 (37.5%)	188 (27.2%)	103 (32.1%)	532 (32.1%)
III	258 (40.2%)	255 (36.8%)	79.0 (24.6%)	592 (35.8%)
IV	143 (22.3%)	249 (36.0%)	139 (43.3%)	531 (32.1%)
**Radio status**				
No	115 (17.9%)	136 (19.7%)	65 (20.2%)	316 (19.1%)
Yes	139 (21.7%)	510 (73.7%)	242 (75.4%)	891 (53.8%)
Missing	388 (60.4%)	46.0 (6.6%)	14.0 (4.4%)	448 (27.1%)

**Figure 2:**
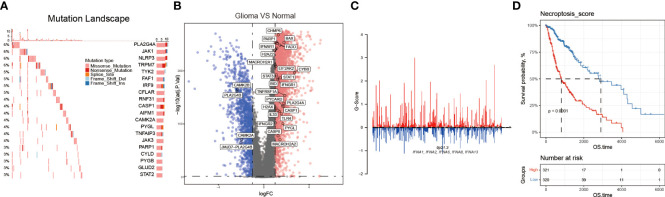
The necroptosis pathway is dysregulated in glioma. **(A)** NRGs mutational landscape. The more frequent mutations included missense and nonsense mutations, splice sites, deletions, and insertions. **(B)** Volcano plot showing some NRGs differentially expressed in glioma and normal brain tissues. **(C)** The CNV, gain, and loss of the NRGs and their distribution on human chromosomes. **(D)** Necroptosis score was calculated by using the GSVA method, and through the K-M curves, activated necroptosis was found associated with poor prognosis (OS).

We first investigated the mutation type and occurrence of the 159 NRGs. The results showed a high occurrence of missense and nonsense mutations, splice sites, deletions, and insertions among the top 20 NRGs (**Figure 2A**). Then, we detected the NRGs’ CNV score using the GISTIC software, and found that the *interferon alpha* (*IFNA*) family genes lost their copy numbers (**Figure 2C**, and **Figures S1A-B**). Next, we combined data from both TCGA and GTEx databases to identify the DEGs between glioma and normal brain tissues using the following parameters: adjusted P value< 0.05, and |log_2_(Fold Change)| > 1. As a result, we screened 29 NRGs with significant differential expressions (**Figure 2B**, and **Figure S1C**). Finally, based on the 159 NRGs and TCGA data, we performed the ssGSEA to calculate the sample enrichment score and combined it with survival analysis, where we found that activation of the necroptosis pathway was associated with poor prognosis (Log-rank, p<0.001) (**Figure 2D**, and **Figures S1D-1E**).

### Establishment and validation of an NRS in glioma

**Figure 3:**
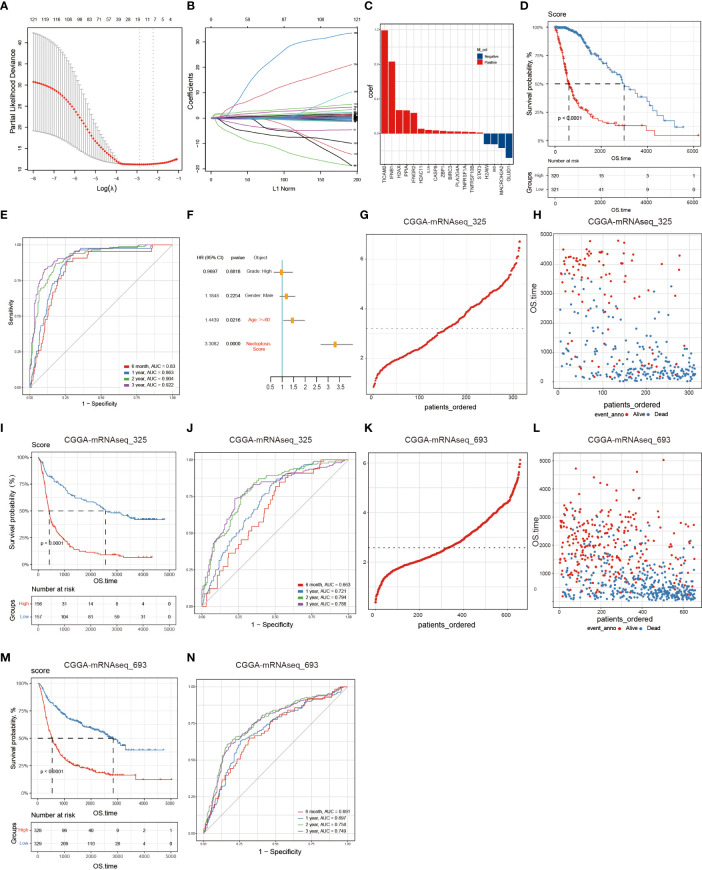
Building and validating the NRS. A total of 126 NRGs were selected *via* the univariate Cox regression and Lasso regression and significantly correlated with prognosis. **(A)** The most optimal parameter selected in Lasso regression by using the 10-fold cross-validation. Red dots indicate the likelihood of deviance values, gray lines represent the standard error (SE), and vertical dot lines correspond to optimal values by minimum criteria, and 1-SE, respectively. **(B)** The Lasso coefficient profile of 126 NRGs, with each curve representing a gene. **(C)** A total of 18 NRGs were incorporated for the NRS construction. **(D)** The survival analysis of the NRS in the TCGA training set. **(E)** Verification of the NRS predictive performance using the Time-ROC analysis. **(F)** Univariate Cox regression analysis illustrates that signature and age were the independent prognostic factor for glioma patients. **(G, H)** The distribution of risk scores of glioma patients in CGGA-mRNA_325 and CGGA-mRNA_693 datasets, respectively. **(I, J)** Numbers of alive and dead patients with different risk scores in CGGA-mRNA_325 and CGGA-mRNA_693 datasets, respectively. **(K, L)** Survival analysis. **(M, N)** Time-ROC analysis.

The above findings reveal the dysregulation of the necroptosis pathway in glioma. To construct an effective necroptosis prognosis signature in glioma, we pre-screened 126 potential NRGs closely associated with prognosis through the univariate Cox regression (**Table S1**). Among these 126 NRGs, we applied the LASSO regression analysis and discovered 18 candidate genes, including the *TIR domain-containing adaptor molecule 2* (*TICAM2*), *interferon beta 1* (*IFNB1*), *H2A.X variant histone* (*H2AX*), *peptidylprolyl isomerase A* (*PPIA*), *interferon-gamma receptor 2* (*IFNGR2*), *H2A clustered histone 11* (*H2AC11*), *interleukin 1 alpha* (*IL1A*), *caspase 8* (*CASP8*), *Z-DNA binding protein 1* (*ZBP1*), *baculoviral IAP repeat containing 3* (*BIRC3*), *phospholipase A2 group IVA* (*PLA2G4A*), *TNF receptor superfamily member 1A* (*TNFRSF1A*), *TNF receptor superfamily member 10 B* (*TNFRSF10B*), *signal transducer and activator of transcription 3* (*STAT3*), *H2A.W histone* (*H2AW*), *BH3 interacting domain death agonist* (*BID*), *macroH2A.2 histone* (*MACROH2A2*), and *glutamate dehydrogenase 1* (*GLUD1*) (**Figures 3A–C**).

Then, Glioma patients in the TCGA training set were classified into low- (n=321) and high- (n=320) risk groups according to the median risk score. The K-M curves showed that compared to the low-risk group, the high-risk group significantly held a poor prognosis (Log-rank test, p<0.0001) (**Figure 3D**). Besides, time-dependent ROC curves showed high sensitivity and specificity for 6 months, 1-, 2-, and 3-year survivals (**Figure 3E**). Finally, we performed the univariate Cox regression analysis on the WHO glioma grades (I-IV), gender, age, and NRS groups, and found that age and risk score were significantly associated with glioma patients’ survival, suggesting that the NRS could serve as a prognosis factor for glioma patients (**Figure 3F**).

Finally, to validate the NRS predictive performance, we assessed the two CGGA datasets (CGGA-mRNAseq_325 and CGGA-mRNAseq_693), which we sorted into both high-risk (mRNAseq_325: n=156, and mRNAseq_693: n=328) and low-risk (mRNAseq_325: n=157, and mRNAseq_693: n=329) groups by using the respective dataset’s median risk score as the cut-off value based on the same calculation formula in the TCGA training set. The K-M curves showed that the high-risk group had a shorter survival period than the low-risk group and the ROC curves proved the predictive effect of the NRS (**Figures 3G–N**).

### Identification of potential signaling pathways and biological processes related to the NRS

**Figure 4:**
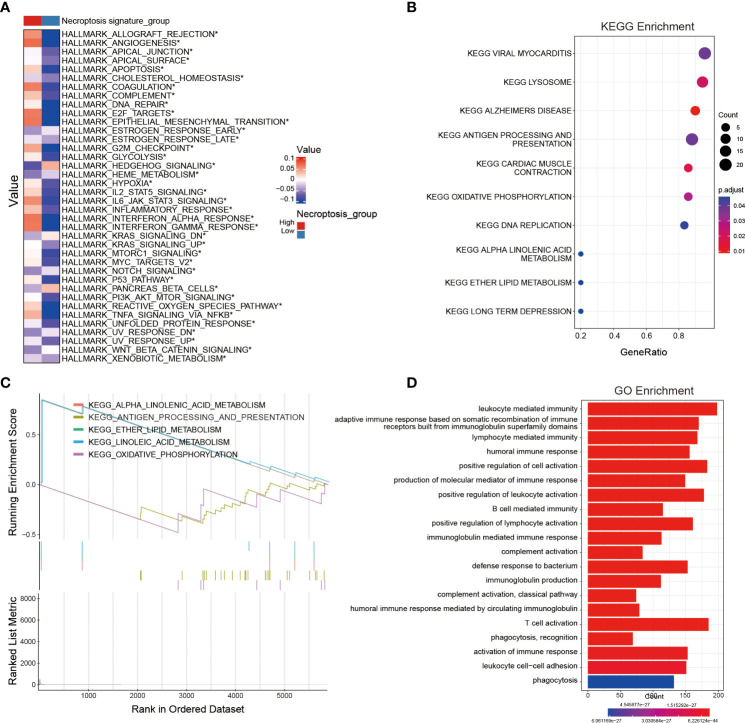
Identification of potential signaling pathways and biological processes associated with the NRS. **(A)** The hallmark pathway enrichment analysis between NRS groups. **(B, C)** KEGG and GSEA enrichment analyses revealing the relevant enrichment pathways of DEGs between NRS groups. **(D)** GO enrichment analysis unveiling the enrichment biological process of DEGs between NRS groups. *: p<0.05.

In order to probe the biological functions related to the NRS, we displayed the differential analysis of the GSVA score on cancer hallmark pathways between different NRS groups, based on the following criteria: FDR< 0.05. Apparently, multiple oncogenic pathways were significantly activated in the high-risk NRS group, such as angiogenesis, hypoxia, KRAS, Notch, PI3K-AKT-mTOR, and WNT-b-catenin. These pathways are closely related to the poor prognosis of glioma patients (**Figure 4A**). In addition, the KEGG and GO enrichment analysis were conducted, based on the DEGs between high- and low-risk groups in the TCGA training set. The KEGG and GSEA enrichments indicated that the signature was related to pivotal biological processes, such as lysosomes, antigen processing and presentation, oxidative phosphorylation, DNA replication, and so on. (**Figures 4B, C**). Besides, the GO enrichment analysis further revealed that a large number of immune-associated biological processes were associated with the NRS, including immune responses mediated by leukocytes, B cells, and T cells (**Figure 4D**). Notably, many immune-inflammatory biological processes and pathways were significantly enriched in three different enrichment methods, suggesting that the difference may be valid in TIME between high- and low-risk NRS groups. Nevertheless, further studies are needed to compare the specific different immune cell infiltration and TIME between the two NRS groups.

### Analysis of tumor immune cell infiltration and chemotherapeutic drug sensitivity between NRS groups

**Figure 5:**
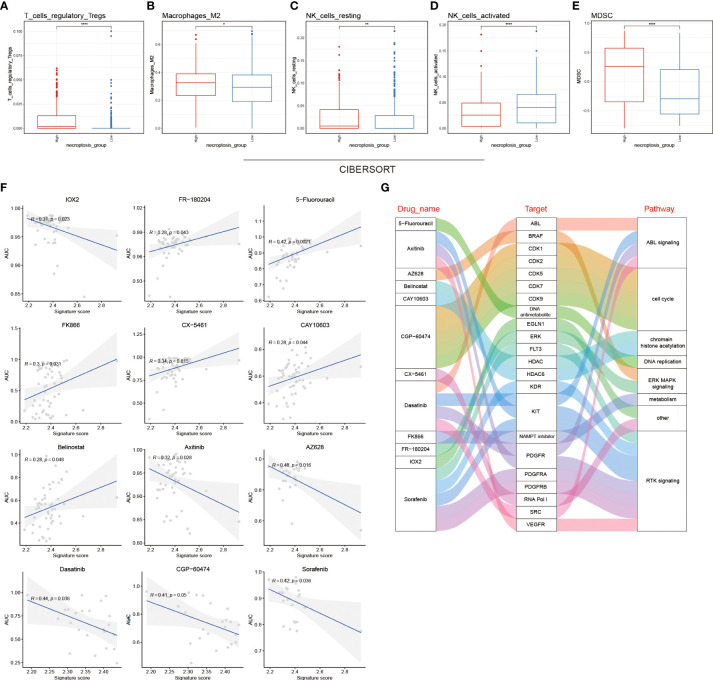
Tumor immune cell infiltration analysis and chemotherapeutic drug sensitivity between NRS groups. Infiltration level analysis of Tregs **(A)**, M2 macrophages **(B)**, resting NK cells **(C)**, activated NK cells **(D)**, and MDSCs **(E)** between NRS groups by using the CIBERSORT algorithm. **(F)** Pearson correlation coefficient between the AUC of 12 chemotherapy drugs and the signature score in the GDSC database. **(G)** Sankey plot showing the specific relationship between the 12 drugs in the GDSC database and their target molecules and pathways. * p<0.05; ** p<0.01 and **** p<0.0001.

Since the occurrence and progression of cancer considerably rely on the TIME, we sought to investigate the relationship between NRS groups and immune cell infiltration. To reach this aim, three algorithms including XCELL, GSVA, and CIBERSORT were performed to calculate the immune cell infiltration between NRS groups. Regulatory T cells (Tregs) negatively regulate the immunoreaction, and previous evidence has demonstrated that the increased grade of glioma is proportional to the number of CD^4+^CD^25+^Foxop3 Treg cells in the peripheral blood of glioma patients, indicating that malignant progression of glioma might be associated with Treg immunosuppression (27, 28). Macrophages are the major immune cells with high plasticity, and two activated forms of macrophages exist including M1 macrophages and M2 macrophages. Cytokines secreted by glioma can activate the STAT3 signaling in macrophages, down-regulate the surface antigens required for the antigen presentation, and up-regulate M2 macrophages-specific antigens like epidermal growth factor (EGF), vascular endothelial growth factor (VEGF), and matrix metallopeptidase (MMPs), which in turn promote the tumor growth and invasion (29–31). Moreover, myeloid-derived suppressor cells (MDSCs) are a group of phenotypic suppressor cells composed of myeloid progenitor cells and myeloid precursor cells in various differentiation stages, such as granulocytes, macrophages, and dendritic cells, known to extensively infiltrate in tumor tissues and exert an immunosuppressive role in the TIME (32, 33). It is also reported that MDSCs’ proportion in peripheral blood mononuclear cells (PBMCs) in GBM patients was evidently higher than that in controls and the accumulation of MDSCs in GBM patients’ peripheral blood may inhibit the immune effects of T cells (34, 35). Combining three immune cell infiltration algorithms, we found that the infiltration of activated M2 macrophages, Tregs, and MDSCs in the high-risk NRS group was significantly higher than that in the low-risk NRS group, which partly explained the effect of the immune cell infiltration on the survival between NRS group (**Figures 5A–E**).

To investigate the chemotherapeutic drug resistance between NRS groups based on the GDSC, CCLE, and CTRP datasets, the R package (oncoPredict) was used to calculate the sensitivity of NRS groups to different chemotherapeutic drugs (**Figures 5F**; **S2A, 2C**). We used the correlation analysis to identify the relationship between the signature score and the AUC of 12 GDSC-derived compounds. IOX2, axitinib, AZ628, dasatinib, CGP-60474, and sorafenib were negatively correlated with the signature score, whereas the remaining compounds (n=6) were positively correlated. Moreover, the 12 compounds were found to inhibit the ABL signaling, cell cycle, histone acetylation, DNA replication, ERK MAPK signaling, cell metabolism, RTK signaling, and other signaling pathways by targeting corresponding core molecules. Specific interactions between drugs, target molecules, and pathways are shown in sankey plot (**Figures 5G** and **S2B, D**).

### Construction of clinical nomograms associated with the NRS

**Figure 6:**
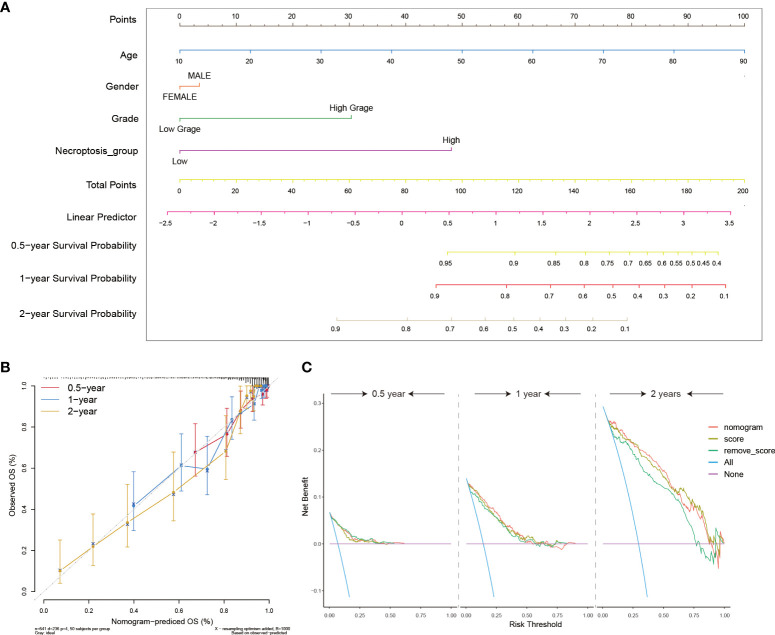
Construction of clinical utility nomograms with the NRS. **(A)** The nomogram included patients’ age, gender, grade, and NRS groups for predicting the 6 months, 1-, and 2-year survival probability of glioma patients. **(B)** Calibration curves showing accuracy and specification of the nomogram and its association with actual clinical effects. **(C)** The DCA analysis of the nomogram for 6-months, 1- and 2-year survival prediction.

To further incorporate the NRS into the clinical diagnosis of glioma prognosis, we drew an intuitional nomogram to thoroughly comprehend the impact of certain risk factors on patient survival (age, gender, and grade) (**Figure 6A**). The calibration curve demonstrated a good agreement between the real observed OS and the ideal nomogram-predicted OS in 6 months, 1- and 2-year survival (**Figure 6B**). Finally, the DCA curves were performed to verify the acceptability of this signature in predicting the probability of glioma patients to survive for 6 months, 1 and 2 years. Our NRS and nomogram had a good predictive performance (**Figure 6C**). All results validated the high precision and practical utility of the NRS.

### Predicting the immunotherapy sensitivity of glioma patients with the present NRS

**Figure 7:**
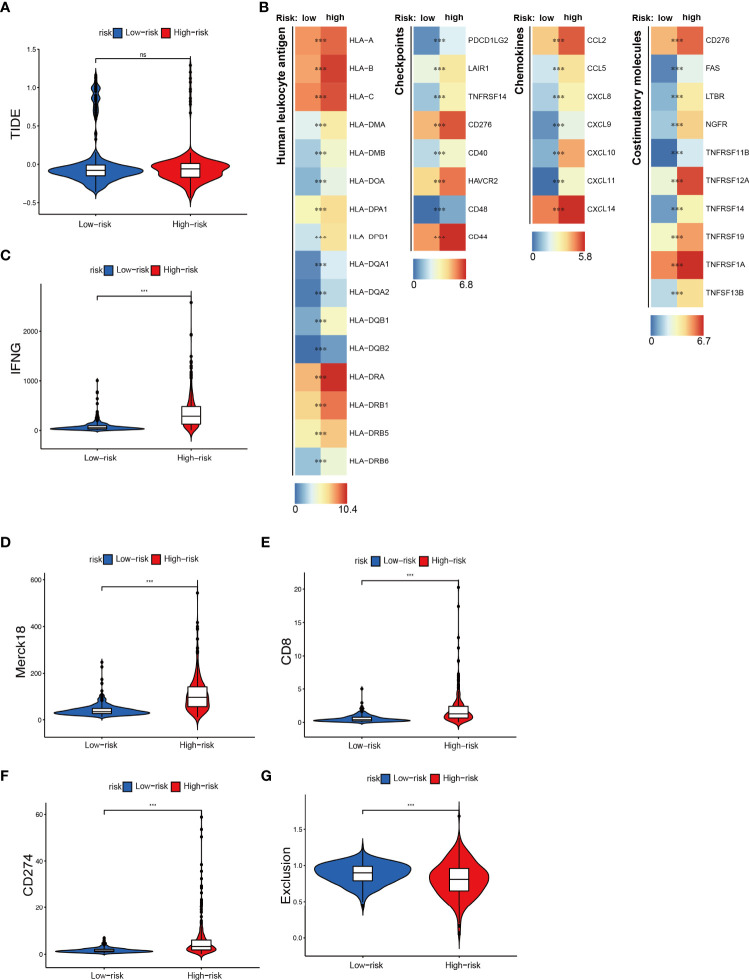
Immunotherapeutic responses exploration between NRS groups. **(A)** The TIDE score in NRS groups. **(B)** Expression of different immune-related indexes between NRS groups. **(C–G)** The expression differences of IFNG, CD8, CD274, and different Exclusion and Merck18 score between NRS groups. *** p<0.001; ns, no significance.

An increasingly number of clinical trials and researches have reported that glioma patients can benefit from related immune checkpoint inhibitors (ICI), CAR-T therapy, and tumor antigen-related vaccines (36–38). Accordingly, we further explored the relationship between NRS groups and the expression of immunotherapy-related targeted molecules. Firstly, we used the TIDE method to evaluate the potential clinical efficacy of immunotherapy in different NRS groups. Among them, the higher the TIDE prediction score, the greater the possibility of immune evasion, indicating that the patient is less likely to benefit from ICI treatment. However, we found there was no significant difference in the TIDE score between the two groups (**Figure 7A**) (39). Next, through the differential analysis, we found that the human leukocyte antigen (HLA), checkpoints, chemokines, and costimulatory molecules were highly expressed in the high NRS group, such as the HLA-B, CD44, CXCL14, and TNFRSF1A (**Figure 7B**). Finally, we found that IFNG, CD8, and CD274 were highly expressed in the high NRS group and the Merck18 score was higher too (**Figures 7C–F**). Meanwhile, the Exclusion score was lower in the high NRS group (**Figure 7G**). The above are representative immunological biomarkers. These results indicated that high NRS group patients may be more likely to benefit from anti-tumor immunotherapy.

### Exploring new necroptosis subtypes from the 18 NRGs in the present NRS

**Figure 8:**
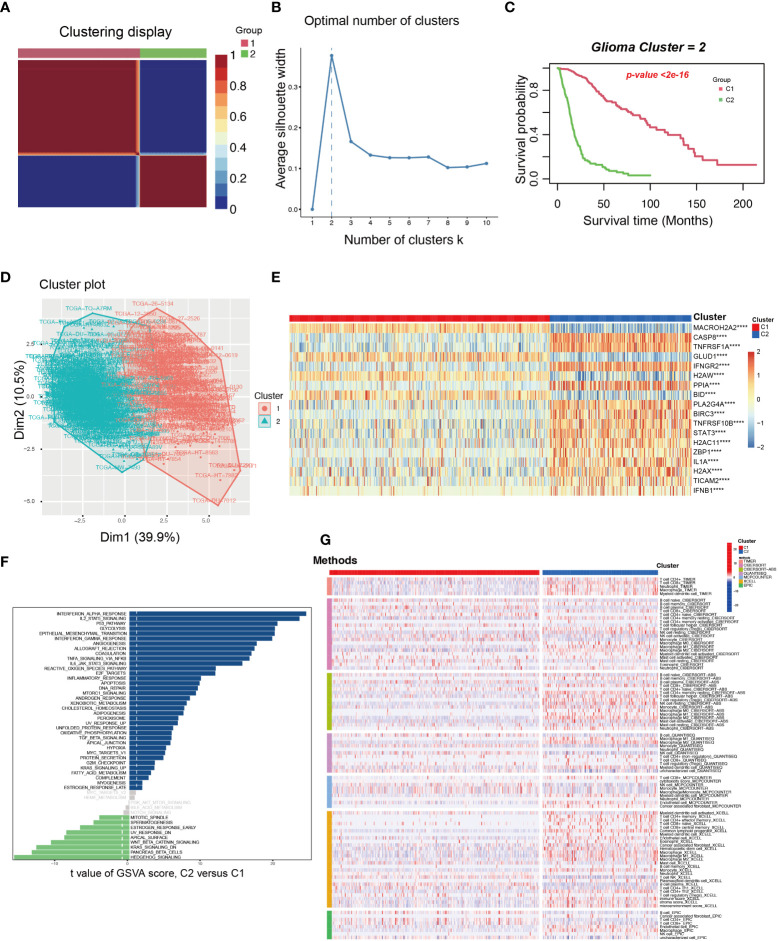
The exploration and assessment of two new subtypes in TCGA glioma patients from the 18 NRGs in the present NRS. **(A)** Based on the CNMF method, the C1 and C2 subtype groups were identified. **(B)** When K=2, the silhouette coefficient is the highest. **(C)** The K-M curves showed that the patients in the C1 group had a higher survival probability than the C2 group. **(D)** PCA analysis illustrated that the two subtypes held different NRGs expression patterns. **(E)** Heatmap showed the 18 NRGs expression in the two subtypes. **(F)** The pathway GSVA score of the two subtypes. **(G)** The heatmap showed 7 algorithms to assess the immune cell infiltration between the two subtypes. ****: p<0.0001.

Based on the 18 NRGs in the present NRS, we identified two new necroptosis-related subtypes in glioma patients by utilizing the consensus non-negative matrix factorization (CNMF) method (Cluster 1 and Cluster 2) (**Figures 8A, B**) (40). Then, the K-M survival analysis showed that C1 held a better prognosis than C2 (Log-rank test) (**Figure 8C**). Dimensionally reduction was employed by Principal Component Analysis (PCA), and we found that the 18 NRGs have evident differential expression patterns (**Figure 8D**). The heatmap showed that the 4 genes, *MACROH2A2*, *GLUD1*, *H2AW*, and *BID* were highly expressed in the C1 group, on the contrary, the other 14 genes were highly expressed in the C2 group (**Figure 8E**). In order to probe the related different pathways of the two subtypes, the GSVA was conducted and we found many immune and inflammatory pathways were significantly up-regulated in the C2 group. (**Figure 8F**). Finally, we used TIMER, CIBERSORT, CIBERSORT-ABS, QUANTISEQ, MCPCOUNTER, XCELL, and EPIC, 7 different algorithms to evaluate the immune cell infiltration between the two subtypes. By comprehensively comparing the 7 methods, we found that the C2 group held a higher immune cell infiltration (**Figure 8G**).

### Identification of potential genes associated with necroptosis in glioma by scRNA-seq

**Figure 9:**
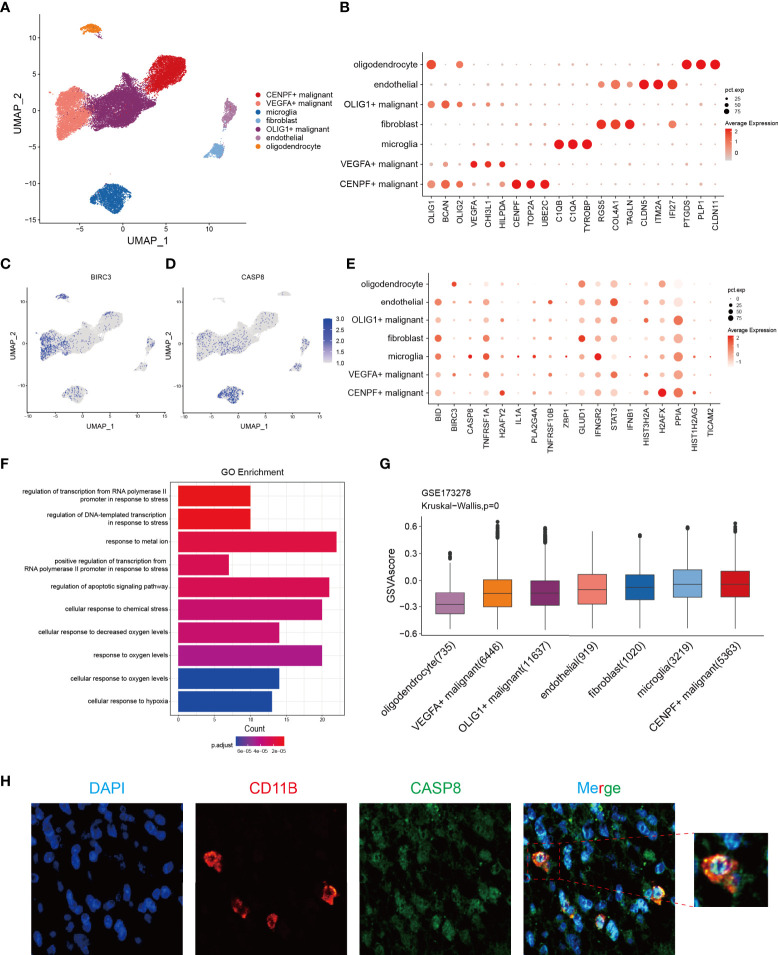
Identification of the potential genes associated with necroptosis in glioma through the scRNA-seq analysis. **(A)** Cells were divided into 7 cell subsets based on the marker gene expression. **(B)** Respective marker genes of the 7-cell subsets. **(C, D)** Expression of *BIRC3* and *CASP8* in different cell subsets, with a specific expression in VEGFA^+^ malignant cells and microglia, respectively. **(E)** Expression levels of 18 NRGs in the NRS from 7 different cell subsets. **(F)** GO enrichment analysis of significant DEGs in VEGFA^+^ malignant cell subsets versus other cell subsets. **(G)** The GSVA score of the necroptosis pathway in each cell subset. **(H)** IFH assay confirmed the localization of CASP8 in microglia (red fluorescent: anti-CD11B, microglia marker; green fluorescent: anti-CASP8; blue fluorescent: DAPI, nuclear).

To resolve the expression patterns of genes in the NRS at a single-cell level, scRNA-seq data in the GSE173278 dataset was selected for analysis. The R-package (Seurat 4.1.0) was used for scRNA-seq analysis and the batch correction between samples was employed by using the harmony algorithm (**Figures S3A-3C**). We used the uniform manifold approximation and projection (UMAP) for dimensionality reduction visualization and a total of 29339 cells were divided into seven categories: CENPF^+^ malignant (n=5363), VEGFA^+^ malignant (n=6446), OLIG1^+^ malignant (n=11637), microglia (n=3219), endothelial cell (n=919), and oligodendrocyte (n=1020) (**Figure 9A**). Corresponding molecular markers were used to identify relevant cell subsets (**Figure 9B**). We analyzed the expression of 18 genes in the NRS in different types of cell subsets (**Figure 9E**), where BIRC3 and CASP8 were specifically expressed in VEGFA^+^ malignant cells and microglia cells, respectively (**Figures 9C, D**). And then, CASP8 expression in microglia was verified by IFH assay in glioma patients’ tissues, and we found a significant colocalization of CASP8 and microglia cell marker CD11B (**Figure 9H**). Because of the specific BIRC3 expression in the VEGFA^+^ malignant cell subset, the biological function of the VEGFA^+^ malignant cell subset was investigated. We analyzed the differences between this subset and the other 6 cell subsets and selected the DEGs for GO enrichment analysis. The results showed that the main enriched biological processes were hypoxia and stress responses (**Figure 9F**). Based on the genes in the NRS, we lastly used the GSVA to evaluate the necroptosis pathway status in each cell, and in general, we found the microglia and CENPF^+^ malignant cells had a higher activated necroptosis status (**Figure 9G**). CASP8, a core molecule located in the necrosome, can selectively trigger apoptosis, necrosis, necroptosis, and inflammatory cell death, such as pyroptosis, depending on its status. CASP8 inhibition in the necroptosis pathway will promote the interaction of RIPK1 with RIPK3, which in turn phosphorylates the downstream molecule MLKL, ultimately leading to cell necroptosis (41). Microglia is a type of macrophage that infiltrates in glioma’s TIME and has two subtypes (M1 and M2), M2 microglia can induce the immunosuppression, invasion, and angiogenesis of glioma by secreting cytokines (42, 43). Therefore, we hypothesized that the activated necroptosis process in microglia might evade the CASP8’s inhibitory effect. These necroptosis microglia, which infiltrate in the glioma TIME induce a immunosuppression, which may be associated with the glioma progression and poor prognosis.

### CASP8 is crucial for glioma progression

**Figure 10:**
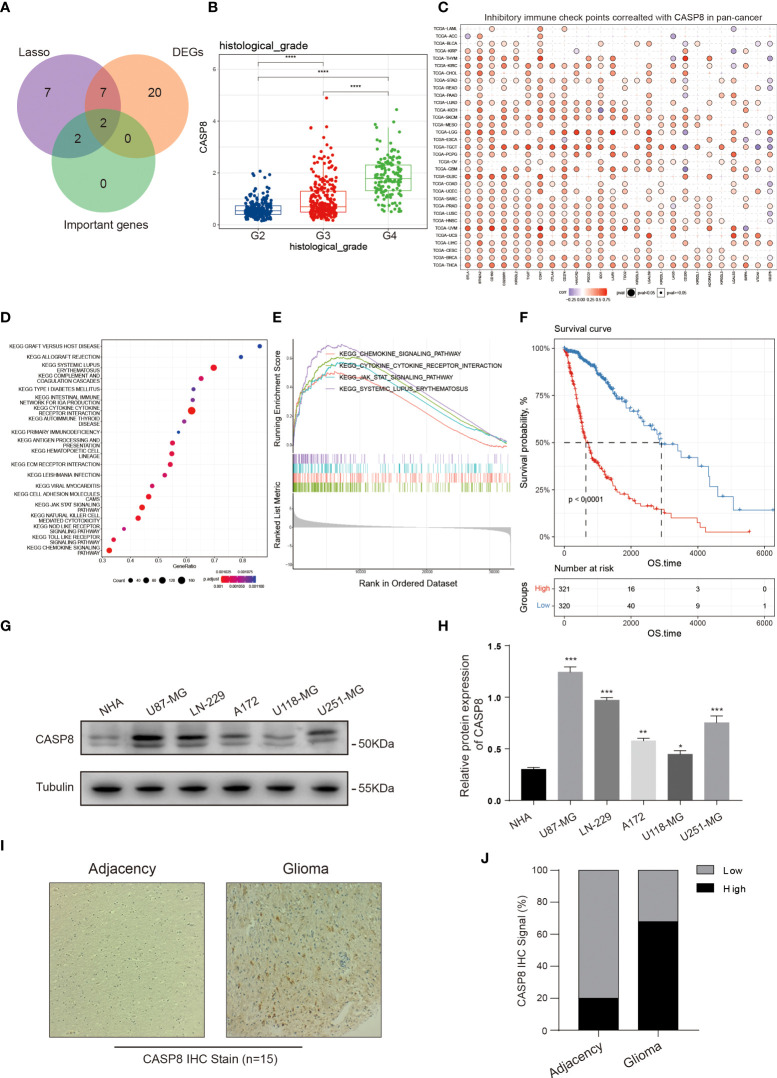
*CASP8* functional exploration. **(A)** Venn plot portraying the common genes in the NRS, DEGs, and random forest analysis. **(B)** The expression of *CASP8* was positively correlated with glioma patient histopathological grades (II-IV). **(C)** Heat map displaying the relationship between various inhibitory immune checkpoints and *CASP8* expression in pan-cancer. **(D, E)** KEGG and GSEA enrichment analyses showing *CASP8*-related signaling pathways and biological processes in glioma. **(F)** The K-M curves suggested that the high *CASP8* expression was associated with low overall survival of glioma patients. **(G, H)** The protein expression of CASP8 in astrocyte NHA and five human glioma cell lines was detected by WB assay, and the protein expression level was quantified in the histogram. The CASP8 protein expression level is lower in glial cell than glioma cell lines. **(I, J)** IHC stain was performed on the 15 glioma patient tissues. Compared with the adjacency, the positive IHC signal of CASP8 was more in the glioma tissues. * p<0.05; ** p<0.01; *** p<0.001 and **** p<0.0001.

A random forest algorithm was employed to identify the most characteristic gene in the NRS gene set. Due to the profound impact on survival in glioma patients, 4 genes, namely *IFNGR2*, *GLUD1*, *PPIA*, and *CASP8* were identified (**Figure S4A**). Both *IFNGR2* and *CASP8* represented the common intersection genes as revealed by the venn plot after combining the DEGs between glioma and normal brain tissues, NRS gene set, and important survival genes from the random forest (**Figure 10A**). Given the key role of CASP8 in regulating cell death process and its high expression in glioma, we further explored the biological function of *CASP8* in glioma at mRNA level, based on the TCGA database. We, that the expression levels of *CASP8* mRNA were considerably increased in different cancer types, including bladder urothelial carcinoma, esophageal carcinoma, stomach adenocarcinoma, etc. (**Figure S4B**). Then, from the K-M curves, an association was obtained between *CASP8* expression and the poor prognosis of glioma patients (**Figures 10F**; **S4C, S4D**). Similarly, a positive correlation between *CASP8* and increased glioma histopathological grades was observed (**Figure 10B**). In addition, *CASP8* expression significantly differed between age groups (Age =<60 VS Age >60), while in gender groups there was no difference (**Figures S4E, F**). Methylation on the gene promoter region, one of the epigenetic modifications, controls gene transcription and expression to a large extent. Thus, based on the TCGA methylation data, we explored the relationship between mRNA and different CASP8 promoter methylation levels [4kb upstream and 100bp downstream of the transcription start site (TSS)] and found the methylation in both of the regions presented an evident negative correlation with *CASP8* expression (**Figures S4G, H**). We also probed the *CASP8* methylation in normal and GBM patients according to the TCGA-GBM cohort. In the primary tumor group, *CASP8* had a lower methylation level than that in the normal group, although the number of samples varied considerably between the two groups (**Figure S4I**). These results about *CASP8* methylation may also reflect the malignant function of *CASP8* in glioma development. Furthermore, a correlation between *CASP8* and chemokines, cytokine receptor interactions, and JAK-STAT signaling pathways was further unveiled using the KEGG and GSEA enrichment analyses (**Figures 10D, E**). Given the association between *CASP8* and immune signaling pathways, we investigated the correlation between *CASP8* and immune checkpoints (**Figures 10C**; **S5A, S5B**) as well as immune cell infiltration (**Figures S5C, D**) by using CIBERSORT and GSVA algorithms. Next, the expression analysis of CASP8 in human astrocyte NHA and 5 immortalized human glioma cell lines (LN-229, U87-MG, A172, U118-MG, and U251-MG) showed that CASP8 expression in astrocytes was lower than that in 5 human glioma cell lines (**Figures 10G, H**). IHC experiment on 15 glioma patient tissues also demonstrated that CASP8 expression in glioma tissues was higher than that in paracancerous tissues (**Figures 10I, J**), further confirming the above analysis of *CASP8* expression in TCGA.

It has been reported that *CASP8* can initiate apoptosis while inhibiting necroptosis (41). However, our results showed that *CASP8* was highly expressed in glioma. Through the ssGSEA score of glioma patients in the TCGA database based on 159 NRGs, we found that the activated necroptosis was closely related to the poor patients’ prognosis, suggesting that necroptosis in glioma might be regulated by other genes than *CASP8*. Therefore, we analyzed the expression patterns of core genes in the KEGG necroptosis pathway between glioma and normal brain tissues (**Figure S6**) and found several upstream genes, such as *TNF receptor superfamily member 1A* (*TNFR1*), *TNF-related apoptosis-inducing ligand receptor* (*TRAILR*), and *interferon production regulator* (*IFNR*) were highly expressed in glioma. Consistently, two vital effector molecules, RIPK1 and RIPK3 were also highly expressed in glioma, suggesting that initiation of necroptosis may be critically regulated by these highly expressed upstream NRGs in glioma.

## Discussion

Mutations in proto-oncogenes and suppressor genes are directly linked to tumor occurrence, which leads to the shutdown of tumor-suppressing signaling and continual activation of tumor-proliferating signaling, resulting in abnormal cell proliferation. At present, with advances in the investigation of the tumor microenvironment (TME) and TIME, researchers have found cancer progression to be related to numerous immunosuppressive cell infiltrations in TIME. Meanwhile, how to inhibit tumor progression by cell death induction has always been a research focus. A variety of small-molecule inhibitors and chemotherapy drugs targeting key regulatory molecules of cell death processes, such as apoptosis, ferroptosis, pyroptosis, and necroptosis, have shown clinical benefits to patients (44, 45). However, the immune suppression caused by the recruitment of immune cells to TIME induced by DAMP release after cell necroptosis has not been fully studied. It has been reported that some drugs can inhibit glioma cell proliferation by inducing necroptosis (46–51). Unfortunately, these findings only rely on *in vitro* studies, which fail to reflect the interaction between glioma necroptosis and TIME. To date, no studies have investigated the relationship between necroptosis and glioma TIME at a single-cell level.

In the present study, we found that some NRGs were highly expressed in glioma and the ssGSEA score suggested an association between activated necroptosis and poor prognosis in glioma patients. This may be consistent with the finding of a previous study that reported an association between RIPK1 overexpression and glioma progression (52). Next, an NRS consisting of 18 NRGs was established and the predictive performance of the NRS was evaluated. Then, the NRS was well validated in two external CGGA datasets, by combining the results, the NRS showed a good performance in assessing the prognosis. Moreover, through the functional enrichment analysis, we found that angiogenesis, KRAS, and WNT signaling pathways were activated in the high-risk group, and KEGG and GO enrichment analysis showed that the DGEs between NRS groups were mainly associated with immune and inflammatory biological functions. Based on these results, we analyzed the different immune cell infiltration to investigate the relationship between necroptosis and TIME in glioma. We found that Tregs, M2 macrophages, and MDSCs were significantly infiltrated in the high-risk group. Combining the TIME analysis and the high expression of NRGs in glioma, we speculated that necroptosis in glioma may enhance the immune response and eliminate own necrotic tumor cells, however, it may probably promote immunosuppression in TIME as well (25, 53–56).

Several studies have reported that injecting necroptotic tumor cells, or engineered cells to specifically overexpress RIPK3 into mouse tumor models leads to killer T cell recruitment to TIME and attack tumor cells. Next, the combination of this therapy with PD-1 immunotherapy could result in long-lasting tumor clearance (57). But, in contrast, some studies have also pointed out that RIPK1 and RIPK3-drived tumor cell necroptosis could induce CXCL1 and SAP130 release that leads to mincle ligation activation and MDSC and M2 macrophage infiltration, which promotes and accelerates immunosuppressive and tumorigenesis, respectively. Our viewpoint is that at an early stage, necroptosis may remove tumor cells, whereas, at a later stage, the immunosuppressive TIME driven by necroptosis contributes to immune escape and results in poor prognosis of glioma patients (8, 25, 58–60). Later, in order to investigate the relationship between immunotherapy and NRS, we analyzed the expression difference of classical immune-associated factors in different NRS groups. Among them, most of the HLA, checkpoints, chemokines, costimulatory molecules, IFNG, CD8, and CD274 were highly expressed in the high-risk group, which showed that the high-risk group may get more chances from the anti-glioma immunotherapy. Such a founding may bring the poor prognosis group some prospect. We discovered two necroptosis-related subtypes by using the CNMF method and assessed them. The two subtypes held different NRGs’ expression patterns, the cluster 2 expressed more immune and inflammatory genes and infiltered a much number of immune cells than did in cluster 1; however, cluster 2 presented a poorer prognosis that may echo the above discussion about the inhibitory glioma TIME associated with necroptosis.

Thereafter, we used the scRNA-seq data to further study the necroptosis in GBM and found through the ssGSEA score that necroptosis was activated in both microglia and certain malignant GBM cell subsets, the necroptotic cells infiltrating in glioma TME may exacerbate the inhibitory TIME. In the end, through differential expression analysis and random forest feature screening, the core intersection gene CASP8 was screened out and the comprehensively functional exploration was conducted on it. We found *CASP8* was significantly overexpressed in a variety of cancers, including glioma. Furthermore, the result indicated that the OS of glioma patient groups with high *CASP8* expression was evidently shortened, and positively correlated with multiple inhibitory immune checkpoints. The three seemingly contradictory findings including the activated necroptosis pathway in glioma patients with poor prognosis and high *CASP8* expression in glioma as well as classical negative regulation of necroptosis by *CASP8* imply that the post-translational modification (PTM) of *CASP8* may play a significant role in necroptotic glioma. As expected, studies have indicated that CASP8 phosphorylation (mediated by ribosomal protein S6 kinase (RSK) recruited into necrosome) at Thr265 can stabilize the necrosome and relieve the inhibitory effect on necroptosis caused by CASP8 (61, 62). However, whether CASP8 expression can be affected by other PTMs, such as ubiquitination, SUMOylation, and glycosylation thereby impacting necroptosis needs further investigation. In addition, the highly expressed *CASP8* in gliomas may be a feedback regulatory mechanism evolved by glioma to avoid necroptosis.

Our study may have some limitations due to the lack of patients’ specific clinical information and follow-up data, and all data derived from public databases. Besides, the difference between gene and protein expression levels might be influenced by several complex biological processes. Furthermore, our hypothesis of which immunosuppression is caused by necroptosis needs further verification. We deem that our study may bring more light to our understanding of how the different necroptosis stages impact the glioma progression.

In conclusion, through analyzing gene expressions and clinical characteristics of glioma patients in the TCGA dataset, we found that certain NRGs harbored mutations and overexpressed in glioma. Besides, a novel NRS was developed, which can effectively assessed the prognosis in glioma patients. Our study further indicated that the activated necroptosis pathway was related to poor prognosis, and multiple immunosuppressive cells were highly infiltrated in the high-risk group. We assumed that the poor prognosis caused by necroptosis may be associated with immunosuppressive TIME. Through scRNA-seq data, we also reported that the necroptosis pathway in GBM and microglial was activated, suggesting that necroptosis in the glioma TIME might promote glioma progression. Our findings may provide guidance for the study of immune escape induced by necroptosis in glioma, and give a new scheme for glioma prognosis prediction. CASP8 high expression seems to be one of the mechanism by which glioma escapes necroptosis and therefore it represents a potential biomarker for glioma prognosis.

## Data availability statement

The original contributions presented in the study are included in the article/**Supplementary Material**. Further inquiries can be directed to the corresponding author.

## Ethics statement

The studies involving human participants were reviewed and approved by the ethics committee of Southwest University. The patients/participants provided their written informed consent to participate in this study.

## Author contributions

HC and PL supervised this research. SW, UAEM, RL, CL, and KW performed the research. SW and UAEM designed the outlines of this research and wrote the manuscript. LD reviewed and revised the manuscript. All authors contributed to the article and approved the submitted version.

## Funding

This research was supported by the Natural Science Foundation of Chongqing (cstc2022ycjh-bgzxm0145, cstc2022ycjh-bgzxm0016, cstc2019jcyj-zdxmX0033), the pilot program of Southwest University (SWU-XDZD22006), and the Fundamental Research Funds for the Central Universities (SWU120054).

## Acknowledgments

Sincerely thanks to the funding support institutions, the members of Prof. Cui’s lab, and assistance of single-cell RNA-seq data analysis from Beijing Gaptech Co., Ltd.

## Conflict of interest

The authors declare that the research was conducted in the absence of any commercial or financial relationships that could be construed as a potential conflict of interest.

## Publisher’s note

All claims expressed in this article are solely those of the authors and do not necessarily represent those of their affiliated organizations, or those of the publisher, the editors and the reviewers. Any product that may be evaluated in this article, or claim that may be made by its manufacturer, is not guaranteed or endorsed by the publisher.
