# Supplementary material for: Combined bulk RNA-seq and single-cell RNA-seq identifies a necroptosis-related prognostic signature associated with inhibitory immune microenvironment in glioma
Source: Front Immunol. 2022 Nov 17;13:1013094. doi: 10.3389/fimmu.2022.1013094 (PMC9713702; doi:10.3389/fimmu.2022.1013094)
Supplement: Supplementary file 1 [file DataSheet_1.docx]

**Supplementary Figure:**


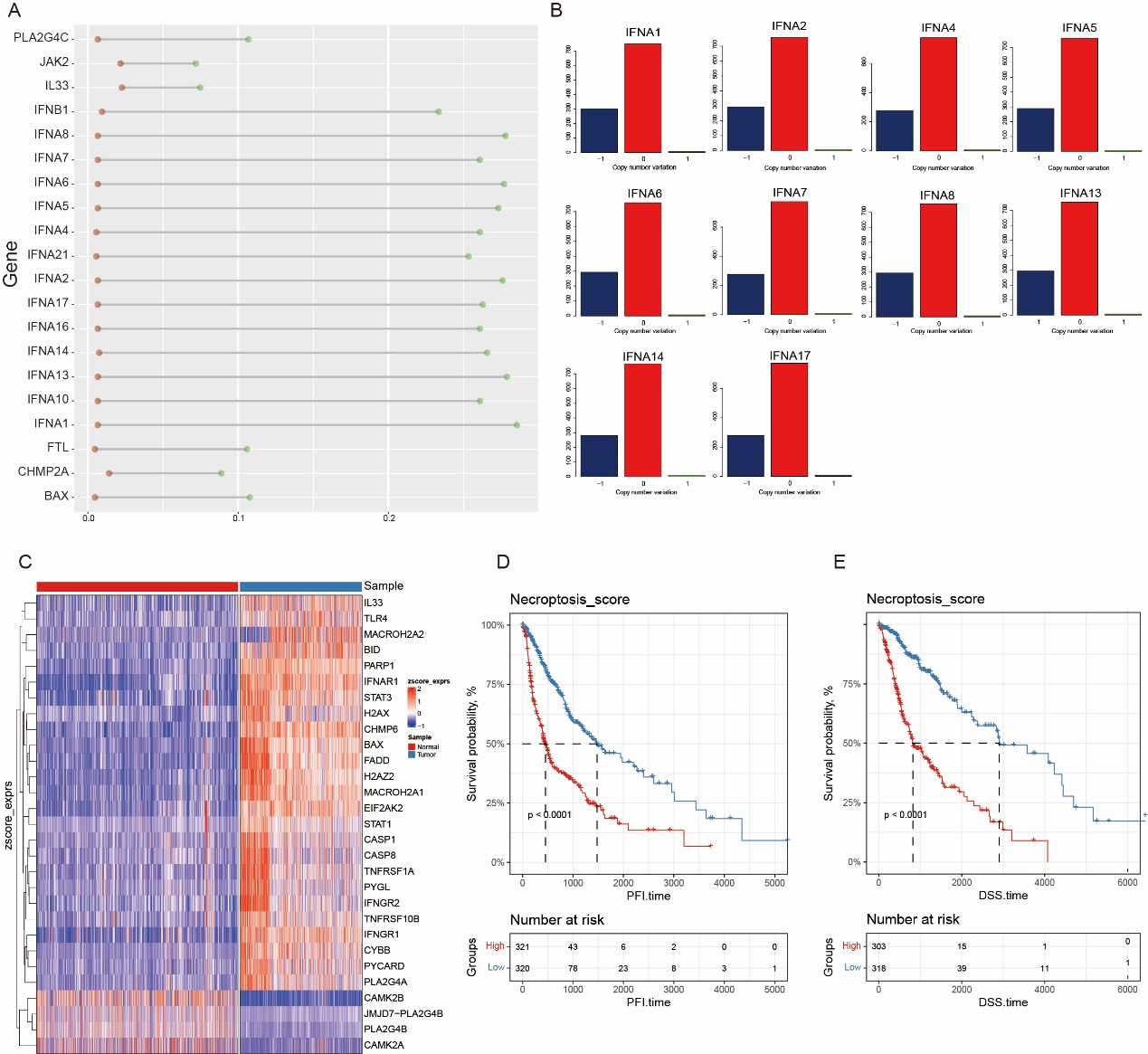


**Figure S1. NRGs are differentially expressed in glioma and harbor CNVs.** (A) The top NRGs with CNVs. Dots represent the proportion of samples with CNVs in the total sample, green dots indicate the loss, and red dots the gain. (B) CNVs of 10 IFNA family genes. (C) Heat map of the 29 differentially expressed NRGs. (D) and (E) Calculation of necroptosis score using the GSVA method. The K-M curves showed that activated necroptosis was associated with poor prognosis (PFI and DSS).


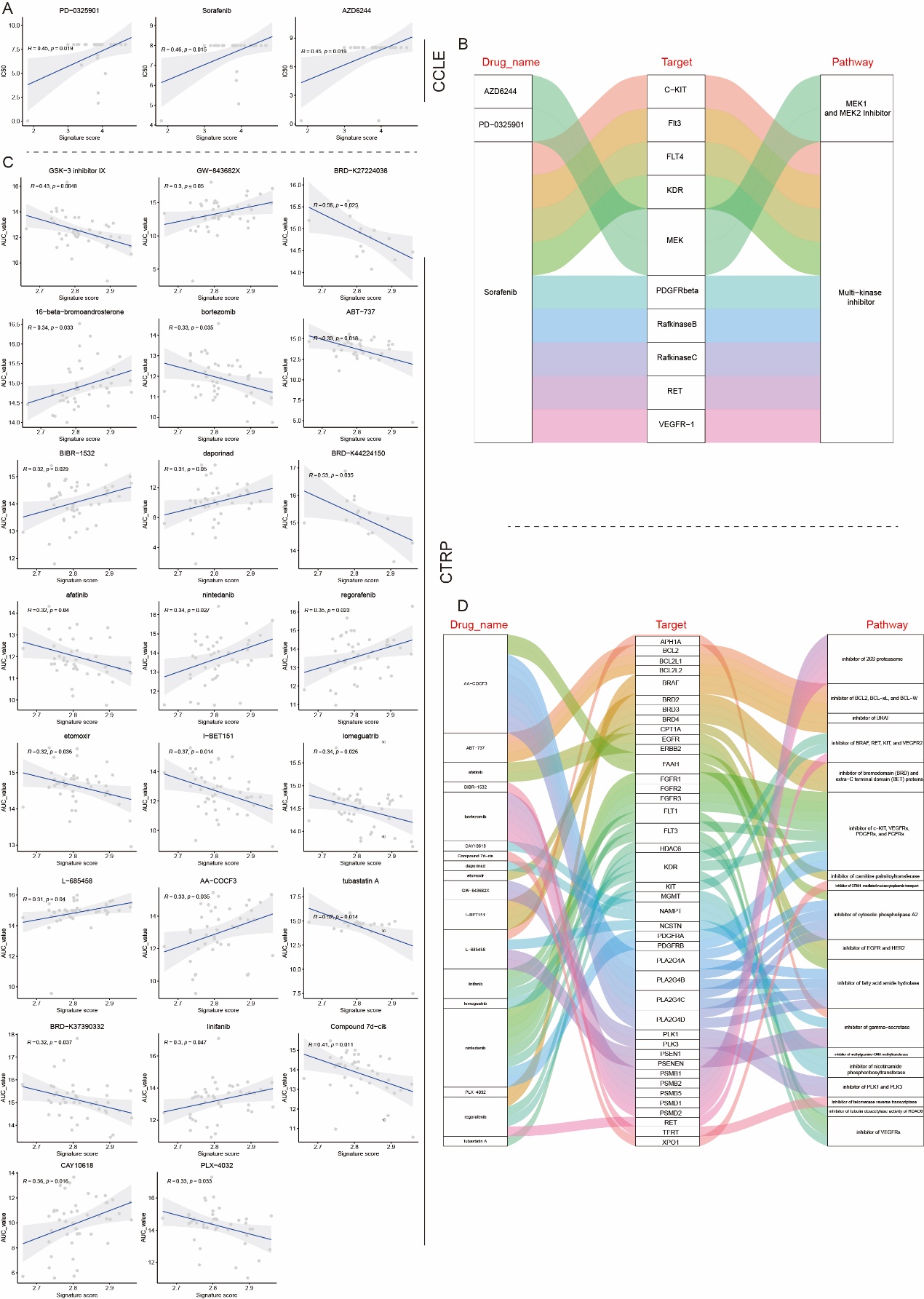


**Figure S2.** (A) The pearson correlation coefficient of the AUC from 3 chemotherapy drugs and the signature score in the CCLE database. (B) Sankey plot showing the specific relationship between the 3 drugs and their target molecules and pathways. (C) and (D) The same analysis as above was performed in the CTRP database.


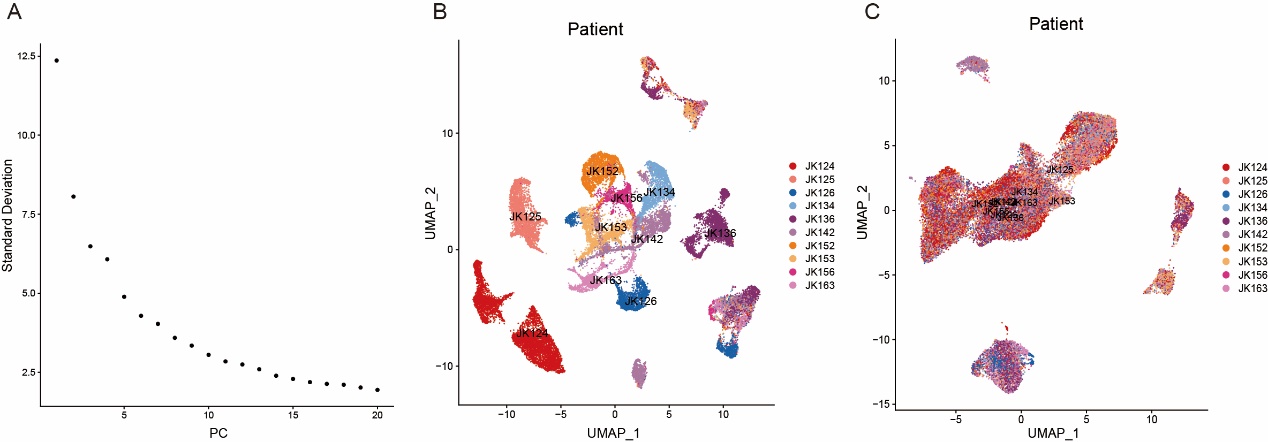


**Figure S3. ScRNA-seq data preprocessing.** (A) The elbow plot was used to determine the number of selected principal components, which are ranked according to the percentage of variance explained by each function. The majority of signals were captured in the top 15 principal components. (B) The nonlinear dimensionality reduction UAMP chart showed a strong batch effect between samples from different patients. (C) The harmony algorithm was used for batch correction, then the batch effect was eliminated.


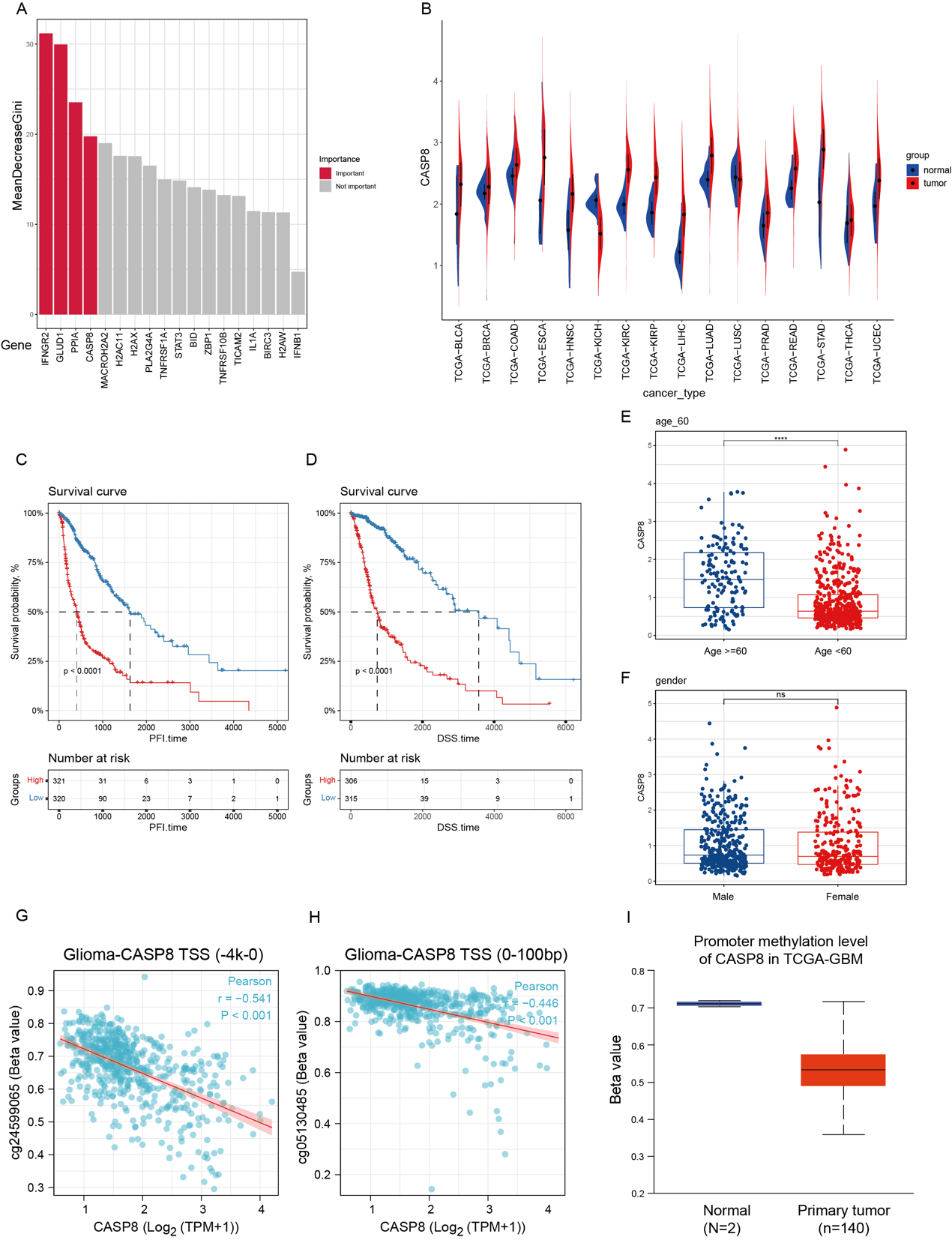


**Figure S4. Comprehensive *CASP8* single gene analysis and functional exploration.** (A) Feature significance was calculated by using a random forest algorithm, and the genes ranked in the top 20% (*IFNGR2*, *GLUD1*, *PPIA*, and *CASP8*) were identified as more important prognosis-related genes in the NRS. (B) Analysis of the TCGA database showed that *CASP8* is highly expressed in various cancer types. (C) and (D) The progression-free interval (PFI) and disease-specific survival (DSS) were compared among the glioma patient group. (E) and (F) *CASP8* expression in a different age (age >= 60 VS age <60) and gender (male VS female) groups from glioma patients, respectively. (G) and (H) The correlation analysis of *CASP8* methylation in different promoter region. (C) The CASP8 different methylation degree in TCGA normal and primary GBM patient group.


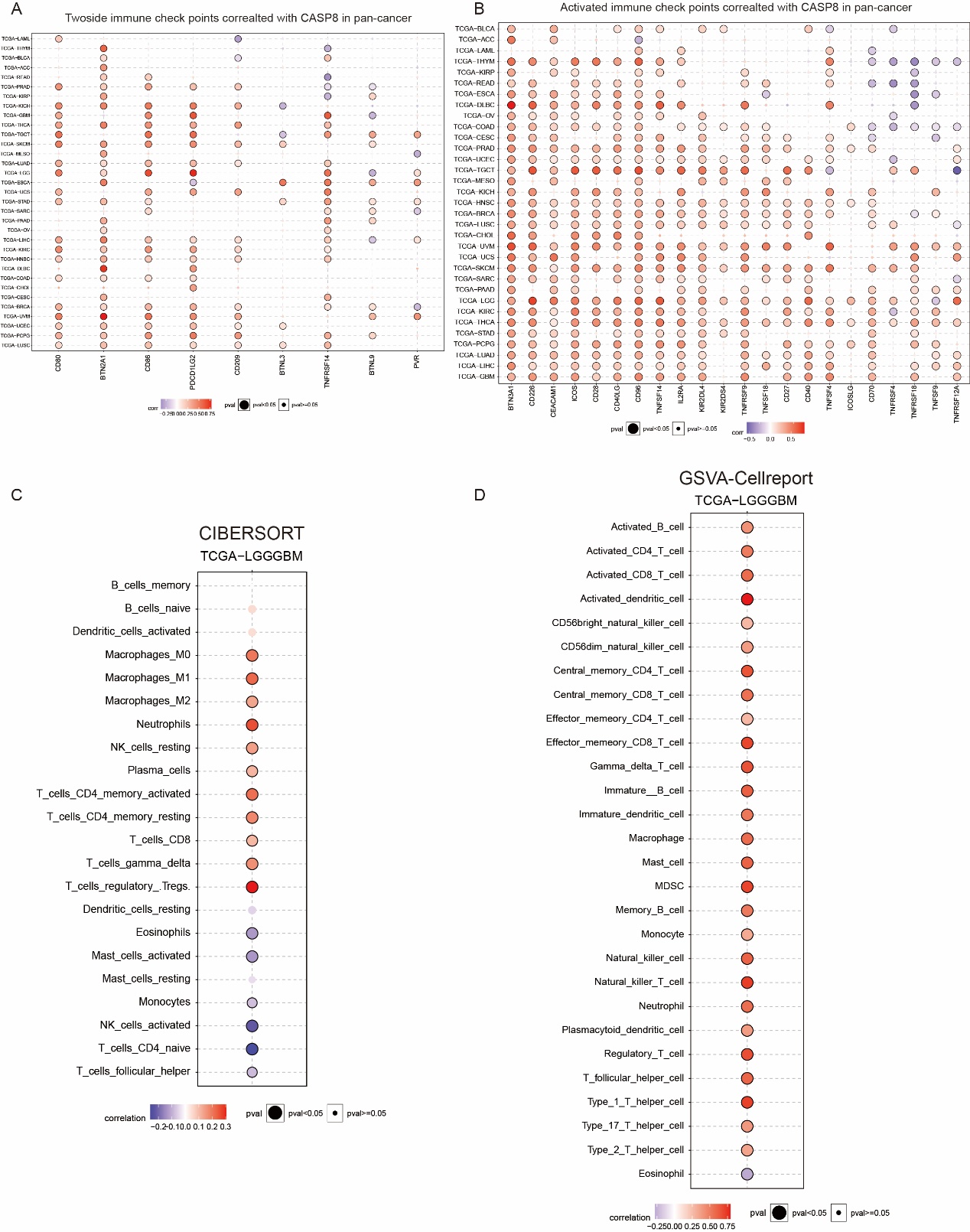


**Figure S5. Immune checkpoints and immune cell infiltration associated with *CASP8* in glioma.** (A) and (B) Correlation between *CASP8* and various two-side and activated immune checkpoints, spearman coefficient. (C) and (D) Correlation of *CASP8* with different immune cell infiltration in glioma assessed by CIBERSORT and GSVA algorithms, respectively.


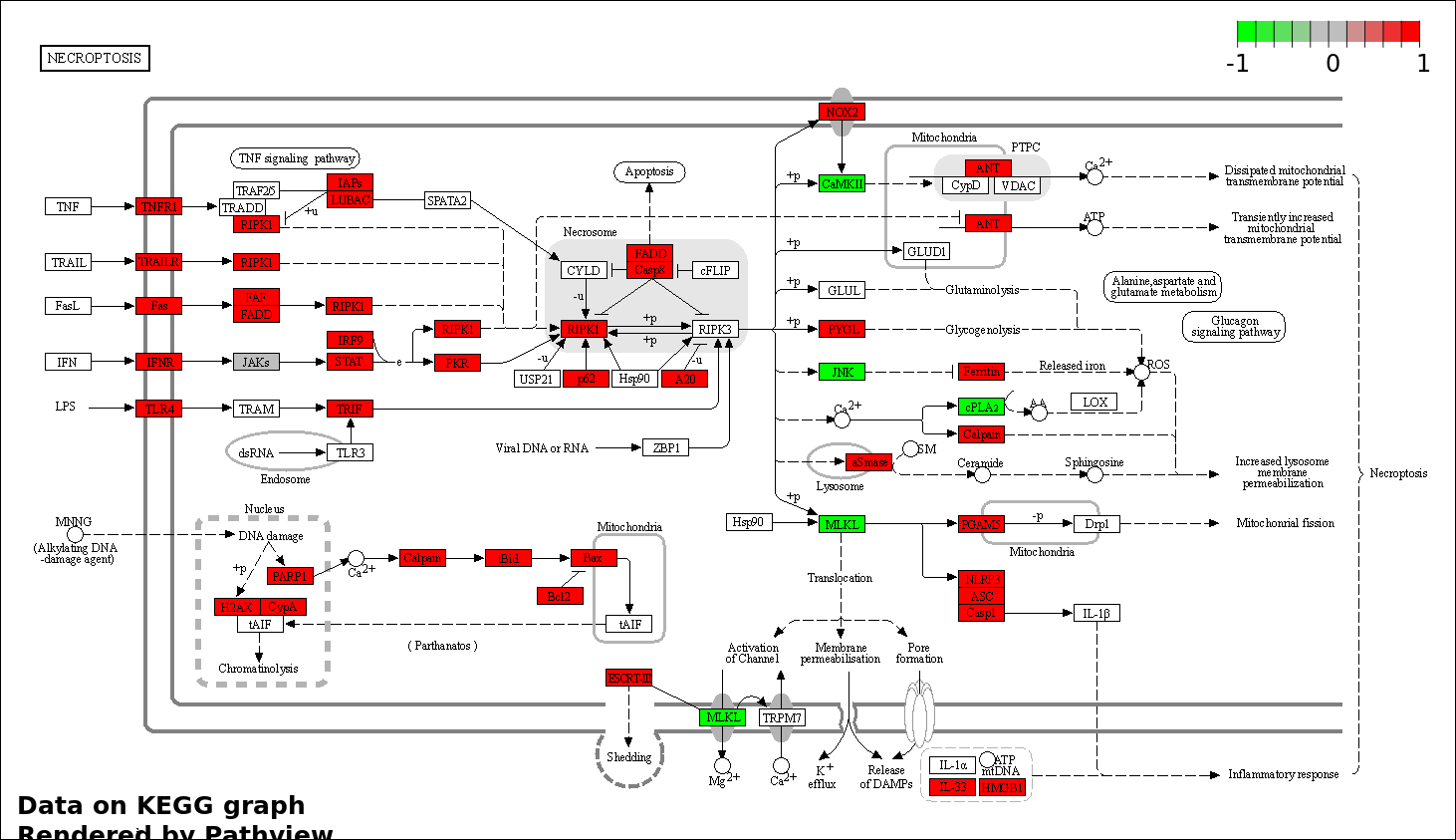


**Figure S6.** Differentially expressed NRGs between glioma and normal brain tissues based on the KEGG necroptosis pathway and the TCGA dataset. Red: high expression in glioma tissues.
